# Modifiable Risk Factors and Residual Risk Following Coronary Revascularization: Insights From a Regionalized Dedicated Follow-Up Clinic

**DOI:** 10.1016/j.mayocpiqo.2021.09.001

**Published:** 2021-12-04

**Authors:** Trevor Simard, Richard G. Jung, Pietro Di Santo, David T. Harnett, Omar Abdel-Razek, F. Daniel Ramirez, Pouya Motazedian, Simon Parlow, Alisha Labinaz, Robert Moreland, Jeffrey Marbach, Anthony Poulin, Amos Levi, Kamran Majeed, Paul Boland, Etienne Couture, Kiran Sarathy, Steven Promislow, Juan J. Russo, Aun Yeong Chong, Derek So, Michael Froeschl, Alexander Dick, Marino Labinaz, Michel Le May, David R. Holmes, Benjamin Hibbert

**Affiliations:** a CAPITAL Research Group, Division of Cardiology, University of Ottawa Heart Institute, Ottawa, Ontario, Canada; b Department of Cellular and Molecular Medicine, University of Ottawa, Ottawa, Ontario, Canada; c Cumming School of Medicine, University of Calgary, Calgary, Alberta, Canada; d Department of Cardiology, Royal Perth Hospital, Perth, Western Australia; e School of Medicine, University of Western Australia, Perth, Western Australia; f Hôpital Cardiologique du Haut-Lévêque, CHU Bordeaux, Bordeaux-Pessac, France; g L’Institut de Rythmologie et Modélisation Cardiaque (LIRYC), Université de Bordeaux, Bordeaux-Pessac, France; h Department of Cardiovascular Diseases, Mayo Clinic College of Medicine, Rochester, Minnesota

**Keywords:** ACS, acute coronary syndrome, CABG, coronary artery bypass grafting, CAD, coronary artery disease, CAPITAL, Cardiovascular And Percutaneous clinical TriALs, DM, diabetes mellitus, HbA1c, hemoglobin A1C, HR, hazard ratio, MACE, major adverse cardiovascular event, MI, myocardial infarction, NSTEMI, non-ST elevation MI, PCI, percutaneous coronary intervention, STEMI, ST elevation MI, UA, unstable angina

## Abstract

**Objective:**

To ensure compliance with optimal secondary prevention strategies and document the residual risk of patients following revascularization, we established a postrevascularization clinic for risk-factor optimization at 1 year, with outcomes recorded in a web-based registry. Although coronary revascularization can reduce ischemia, medical treatment of coronary artery disease (CAD) remains the cornerstone of ongoing risk reduction. While standardized referral pathways and protocols for revascularization are prevalent and well studied, post-revascularization care is often less formalized.

**Patients and Methods:**

The University of Ottawa Heart Institute is a tertiary-care center providing coronary revascularization services. From 2015 to 2019, data were prospectively recorded in the CAPITAL revascularization registry, and patient-level procedural, clinical, and outcome data are collected in the year following revascularization. Major adverse cardiovascular event (MACE) was defined as death, myocardial infarction, unplanned revascularization, or cerebrovascular accident. Kaplan-Meier curves were generated to evaluate time-to-event data for clinical outcomes by risk-factor management, and comparisons were performed using log-rank tests and reported by hazard ratio (HR) and 95% confidence intervals (CIs).

**Results:**

A cohort of 4147 patients completed 1-year follow-up after revascularization procedure that included 3462 undergoing percutaneous coronary intervention (PCI), 589 undergoing coronary artery bypass graft (CABG), and 96 undergoing both PCI and CABG. In the year following revascularization (median follow-up 13.3 months—interquartile range [IQR]: 11.9-16.5) 11% of patients experienced MACE, with female patients being disproportionately at risk. Moreover, 47.7% of patients had ≥2 risk factors (diabetes, dyslipidemia, overweight, active smoker) at the time of follow-up, with 45.0% of patients with diabetes failing to achieve target hemoglobin (Hb) A1c, 54.8% of smokers continuing to smoke, and 27.1% of patients failing to achieve guideline-directed lipid targets.

**Conclusion:**

Patients who have undergone revascularization procedures remain at elevated risk for MACE, and inadequately controlled risk factors are prevalent in follow-up. This highlights the need for aggressive secondary prevention strategies and implementation of programs to optimize postrevascularization care.

Risk factors for adverse cardiovascular events are well established. Conversely, protective factors including healthy diet and exercise are known to mitigate risk.^1^ These form the baseline of care irrespective of whether the patient has undergone revascularization or not. Accordingly, secondary prevention strategies are vital to optimize a patient’s risk profile and to minimize the risk of adverse events following coronary revascularization. Despite these efforts, coronary artery disease (CAD) continues to be a leading cause of morbidity and mortality.^2^^,^^3^

Advances in revascularization care include both changes in medical therapy^4^, ^5^, ^6^ and procedural technology and technique.^7^, ^8^, ^9^, ^10^, ^11^ Contemporary revascularization is more commonly performed via percutaneous coronary intervention (PCI) than coronary artery bypass grafting (CABG), with the mode of revascularization selected based on clinical presentation, disease complexity, and comorbidity burden.^12^^,^^13^ For PCI, improvements in stent design and techniques (eg, imaging and fractional flow reserve) has reduced repeat revascularization rates,^14^, ^15^, ^16^, ^17^, ^18^ although this has not translated to reduced rates of death or myocardial infarction (MI).^15^ Similarly, CABG reports annualized graft failure rates of < 5% for arterial and up to 25% for venous conduits, with pooled data suggesting a benefit of arterial conduits to reduce MI.^19^ However, irrespective of the mode of revascularization, long-term outcomes are most affected by risk-factor modification and medical therapy. Indeed, cumulative rates of death or nonfatal MI post-PCI approach 17% at 6 years postrevascularization without plateauing.^15^ Indeed, following CABG or PCI major adverse cardiovascular events (MACE)—death, MI, stroke, or repeat revascularization—rates approach 20% to 28% at 3 years, with a 24% to 27% mortality rate at 10 years,^20^ highlighting the need for ongoing risk-factor control.^21^

The first year postrevascularization represents the highest-risk period for patients with coronary artery disease (CAD).^21^ Although considerable resources and research have established optimal pathways to enable patients to achieve timely revascularization,^22^ protocols for optimal care thereafter are not as well established. Accordingly, we established a standardized postrevascularization clinic, whereby all patients undergoing revascularization procedures undergo protocolized assessment in the year following their revascularization procedures. The purpose of this program is to assess their risk-factor management uniformly and to implement optimal secondary prevention strategies. Herein, we evaluate the effectiveness of established care pathways on risk-factor management during the first 12 months after coronary revascularization.

## Methods

### Study Population and Data Collection

The University of Ottawa Heart Institute is a large tertiary-care center providing the sole coronary revascularization services to more than 1.2 million people in the capital region of Canada, including an established primary PCI program for patients with ST-elevation myocardial infarction (STEMI) with a hub-and-spoke model for peripheral community centers.^22^ Our center includes an established cardiac rehabilitation program with integrated physical therapy, dietary, psychosocial, and smoking-cessation programs offered to all revascularization patients.^23^, ^24^, ^25^, ^26^ All patients undergoing revascularization have their data prospectively recorded in the CArdiovascular Percutaneous Intervention TriAL (CAPITAL) revascularization registry, a web-based registry that captures more than 1200 clinical data points on background and procedural factors related to revascularization. This registry also includes a subset of patients with samples collected in the CAPITAL Biobank to gain insights into novel biomarkers in patients undergoing invasive angiography.^27^, ^28^, ^29^, ^30^ Comorbidities are documented at the time of preprocedural assessment by the clinician, with hypertension and dyslipidemia determined based on existing diagnoses using guideline recommendations or presence of dedicated medical therapy.^31^^,^^32^ Diabetes mellitus (DM) was determined from previous history, presence of DM agents, or a hemoglobin A1c (HbA1c) ≥6.5% at presentation with types delineated as type 1, type 2 non–insulin-dependent, type 2 insulin-dependent. Medications were recorded from medical reconciliation lists. Acute coronary syndrome (ACS) was subclassified as STEMI, non–ST-segment elevation myocardial infarction (NSTEMI), and unstable angina (UA).^33^ CAD was defined as ≥50% visual stenosis of an epicardial artery documented at the time of invasive angiography. Revascularization procedures include patients who underwent angiography at our center and subsequently underwent PCI or CABG. Subjects with multiple invasive angiograms were included once for the purposes of analysis with their first invasive angiogram representing the index event and subsequent invasive angiograms recorded and used to identify revascularization events. The study was approved by Ottawa Health Science Network Research Ethics Board (OHSN-REB #20190224-01H) to evaluate clinical outcomes following revascularization.

### Follow-Up Protocol

**Figure 1:**
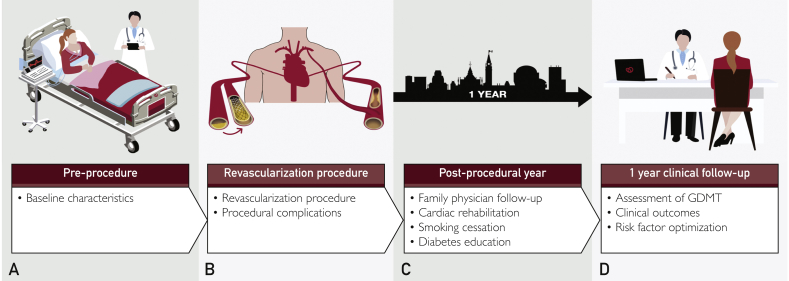
Postrevascularization clinic workflow. A, Before the procedure, all baseline characteristics are recorded in the CAPITAL revascularization registry. B, Procedural data and complications are recorded following the completion of revascularization. C, Following revascularization, patients underwent primary care physician follow-up, cardiac rehabilitation, smoking cessation, and diabetes management as appropriate. D, One-year clinical follow-up performed with assessment of guideline-directed medical therapy, risk-factor optimization, and clinical outcomes.

After revascularization, per our local process, patients assumed established, predefined cardiac rehabilitation protocols and follow-up with primary care physicians. At 1-year post-revascularization, they were contacted to return for clinical follow-up with reassessment of lipid profile and glycemic control at that time. Patients who were unable to return for in-person follow-up underwent telephone follow-up as possible. In-person follow-ups were completed by physicians performing standardized assessments with a focus on cardiovascular risk factor management and optimization of relevant medical therapy (Figure 1).

### Clinical Outcomes

Predefined clinical parameters were recorded at the time of angiography and at follow-up. Risk-factor management was dichotomized following clinical guidelines. Modifiable risk factors available were predefined as DM, smoking, lipid levels and body mass index (BMI) status. Glycemic control was defined as HbA1c ≤ 7.0% in those with DM. Smoking status was dichotomized as active or not active at the time of follow-up, with subsets including “quit but relapsed” and “never quit since index case” to reflect changes in the year following revascularization. Low-density lipoprotein (LDL) levels were reassessed with adequate levels set as per guidelines targets of <1.8 to 2.0 mmol/L.^34^, ^35^, ^36^ Patient body-mass indices (BMIs) were recorded at the time of angiography and again at 1-year follow-up. Baseline and follow-up BMIs were grouped into underweight (<18.5 kg/m^2^), normal (18.5 to 24.9 kg/m^2^), and overweight to obese (≥25.0 kg/m^2^). Significant weight loss was defined as follow-up weight that was ≥10% less than the body weight at the time of index case;^37^ MACE was assessed at 1 and 12 months, defined as a composite of death, MI, stroke (as per neurologist assessment or hemorrhagic cerebrovascular event with confirmatory imaging), or any repeat unplanned revascularization procedure, individual components of this outcome are reported separately. Patients who died before follow-up assessment were excluded from risk-factor analysis.

### Statistical Analysis

Continuous variables are reported as mean ± standard deviation or median ± interquartile range (IQR). Categorical variables were compared using the χ^2^ or Fisher’s exact tests, and continuous variables were compared by Student's *t*-tests or Mann-Whitney U tests, as appropriate. Kaplan-Meier curves were generated to evaluate time-to-event data for clinical outcomes by risk-factor management and comparisons were performed using log-rank tests. Patients were censored after the first occurrence of MACE. Hazard ratios (HRs) with 95% confidence intervals (CIs) were calculated using Cox regression. Odds ratios (ORs) with 95% CI were calculated to evaluate the association between modifiable risk factors at time of follow-up. All statistical analyses were performed using SAS v9.4 (SAS Institute, Inc, Cary, North Carolina), and all figures were created using GraphPad Prism v8 (GraphPad Software, San Diego, California). *P*<0.05 was considered statistically significant.

## Results

**Figure 2:**
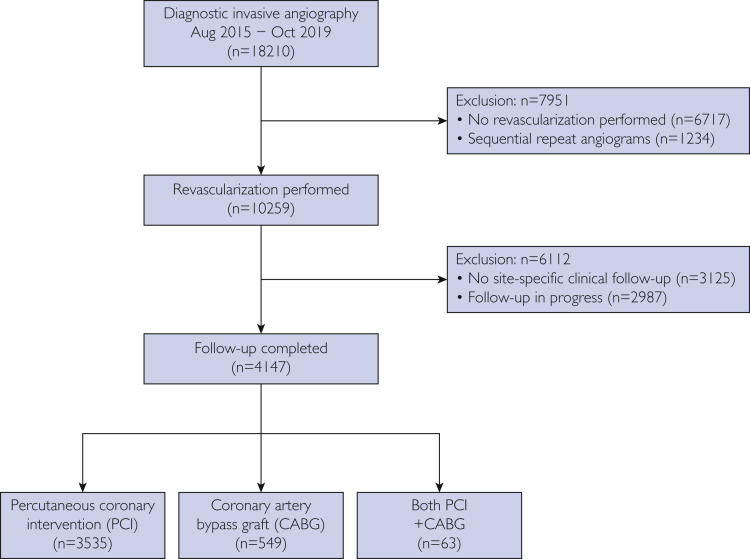
Patient flow.

From August 2015 to October 2019, 18,210 coronary angiograms were performed; 1234 were repeat procedures, and 6717 did not undergo revascularization. Of 18,210 patients, 10,259 went on to revascularization, 2987 of whom were excluded, as 12 months had not elapsed since their procedure at the time of analysis; 3125 patients elected for routine follow-up outside of the revascularization clinic. Thus, outcome data of interest were available for 4147 patients, 3462 of whom underwent PCI; 589 underwent CABG, and 96 had staged procedures with both PCI and CABG (Figure 2).

### Patient Characteristics

The baseline characteristics of patients are summarized in Table. The mean age was 65.8±11.8 years, and 1068 patients (25.8%) were women. The mean BMI was 29.0±5.7 kg/m^2^. Risk factors included type 2 DM (25.7%), active smoking (19.9%), dyslipidemia (57.4%), hypertension (60.3%), and family history of CAD (14.3%). At baseline, medical therapy in the cohort included aspirin (90.8%), P2Y_12_ inhibitors (91.1%), statins (69.6%), angiotensin converting-enzyme inhibitors or angiotensin receptor blockers (ACEi/ARB) (41.5%), and beta blockers (45.6%). The indication for revascularization varied, 64.4% presented with ACS (28.0% STEMI, 36.4% NSTEMI/UA), whereas 24.2% of patients had stable CAD or chronic coronary syndrome.^38^ Female patients were older, with a higher incidence of hypertension, diabetes, and smoking and less likely to have had previous PCI or MI. Female patients presented more commonly with ACS, underwent more femoral access, and were more likely to be revascularized by PCI and, less likely, CABG compared with male patients. In follow-up, female patients were less likely to be taking aspirin, ACEi or ARBs, and statins.

**Table:** Baseline characteristics

	Total (n=4147)	Male (n=3079)	Female (n=1068)	P-value
Age: years, mean ± SD	65.8±11.8	64.5±11.5	69.5±11.8	<0.0001
Sex (female): no. (%)	1068 (25.8)	-	-	-
BMI: kg/m^2^, mean ± SD	29.0±5.7	28.9±5.2	29.2±7.0	0.17
Hypertension: no. (%)	2502 (60.3)	1763 (57.3)	739 (69.2)	<0.0001
Dyslipidemia: no. (%)	2381 (57.4)	1759 (57.1)	622 (58.2)	0.53
Diabetes: no. (%)				
Type 1	26 (0.6)	16 (0.5)	10 (0.9)	0.14
Type 2	1065 (25.7)	756 (24.6)	309 (28.9)	0.004
Smoking: no. (%)				0.0005
Never	2402 (57.9)	1731 (56.2)	671 (62.8)	
Remote (quit >1 month ago)	920 (22.2)	701 (22.8)	219 (20.5)	
Active	825 (19.9)	647 (21.0)	178 (16.7)	
Previous history: no. (%)				
PCI	745 (18.0)	601 (20.1)	144 (13.9)	<0.0001
MI	635 (15.3)	500 (16.7)	135 (13.0)	0.005
CABG	210 (5.1)	166 (5.5)	44 (4.2)	0.1
PAD	193 (4.7)	134 (4.4)	59 (5.5)	0.12
CVA	178 (4.3)	122 (4.0)	56 (5.2)	0.08
Heart failure	138 (3.3)	96 (3.1)	42 (3.9)	0.2
Medications, baseline: no. (%)				
ASA	3767 (90.8)	2779 (90.3)	988 (92.5)	0.03
P2Y12	3776 (91.1)	2780 (90.3)	996 (93.3)	0.003
ACEi/ARB	1722 (41.5)	1265 (41.1)	457 (42.8)	0.33
Beta blocker	1889 (45.6)	1390 (45.1)	499 (46.7)	0.37
Calcium channel blocker	403 (9.7)	288 (9.4)	115 (10.8)	0.18
Statin	2888 (69.6)	2136 (69.4)	752 (70.4)	0.52
PPI	492 (11.9)	319 (10.4)	173 (16.2)	<0.0001
Investigations, baseline				
Creatinine: mean +/– SD (mmol/L)	93.7±68.1	96.4±67.1	86.2±70.3	<0.0001
CrCl: mL/min, mean +/– SD	91.1±40.2	96.2±39.5	77.0±38.8	<0.0001
LVEF (n=1138)				0.06
Normal	813 (71.4)	564 (69.4)	249 (76.6)	
>45%	134 (11.8)	104 (12.8)	30 (9.2)	
30%-45%	139 (12.2)	109 (13.4)	30 (9.2)	
<30%	52 (4.6)	36 (4.4)	16 (4.9)	
Mitral valvulopathy (≥moderate)	53 (1.3)	30 (1.0)	23 (2.2)	0.003
Aortic valvulopathy (≥moderate)	108 (2.6)	72 (2.3)	35 (3.4)	0.07
Procedural details				
Indications: no. (%)				
Acute coronary syndrome	2670 (64.4)	1949 (63.3)	721 (67.5)	0.01
STEMI	1160 (28.0)	863 (44.3)	297 (41.2)	0.15
NSTEMI/unstable angina	1510 (36.4)	1086 (55.7)	424 (58.8)	
Staged PCI	311 (7.5)	242 (7.9)	69 (6.5)	0.13
Stable CAD	1005 (24.2)	757 (24.6)	248 (23.2)	0.37
Shock	53 (1.3)	34 (1.1)	19 (1.8)	0.09
Access: no. (%)				<0.0001
Radial	3231 (77.9)	2454 (79.7)	777 (72.8)	
Femoral	910 (21.9)	621 (20.2)	289 (27.1)	
Revascularization method: no. (%)				
PCI	3535 (85.2)	2591 (84.2)	944 (88.4)	0.001
CABG	549 (13.2)	437 (14.2)	112 (10.5)	0.002
Both	63 (1.5)	51 (1.7)	12 (1.1)	0.22
Medications, follow-up: no. (%)				
ASA	3393 (81.8)	2579 (83.8)	814 (76.2)	<0.0001
P2Y12	2360 (56.9)	1749 (56.8)	611 (57.2)	0.82
ACEi/ARB	2529 (61.0)	1913 (62.1)	616 (57.7)	0.01
Beta blocker	2678 (64.6)	1998 (64.9)	680 (63.7)	0.47
Calcium channel blocker	518 (12.5)	372 (12.1)	146 (13.7)	0.18
Statin	3484 (84.0)	2619 (85.1)	865 (81.0)	0.002
DAPT score ≥2: no. (%)	1354 (32.7)	1039 (33.7)	315 (29.5)	0.01

ACEi/ARB, angiotensin-converting enzyme inhibitor/angiotensin-receptor blocker; ASA, acetylsalicylic acid; BMI, body mass index; CABG, coronary artery bypass graft; CAD, coronary artery disease; CrCl, creatinine clearance; CVA, cerebrovascular accident; DAPT, dual antiplatelet therapy; LVEF, left ventricular ejection fraction; MI, myocardial infarction; NSTEMI, non–ST-elevation MI; PAD, peripheral arterial disease; PCI, percutaneous coronary intervention; PPI, proton pump inhibitor; SD, standard deviation; STEMI, ST-elevation MI.

### Clinical Outcomes

**Figure 3:**
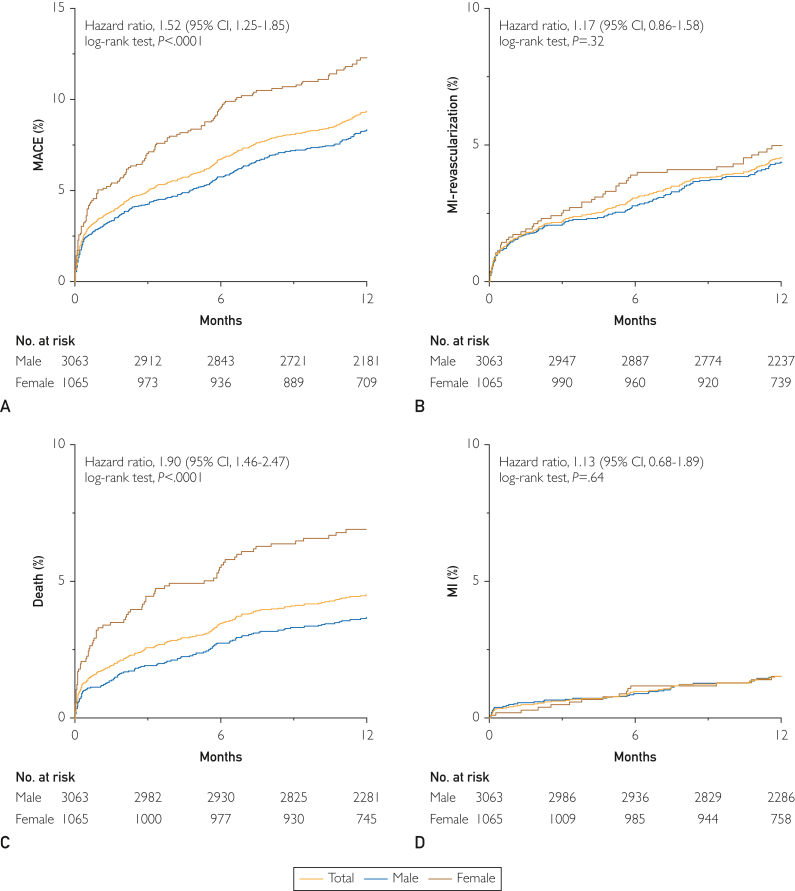

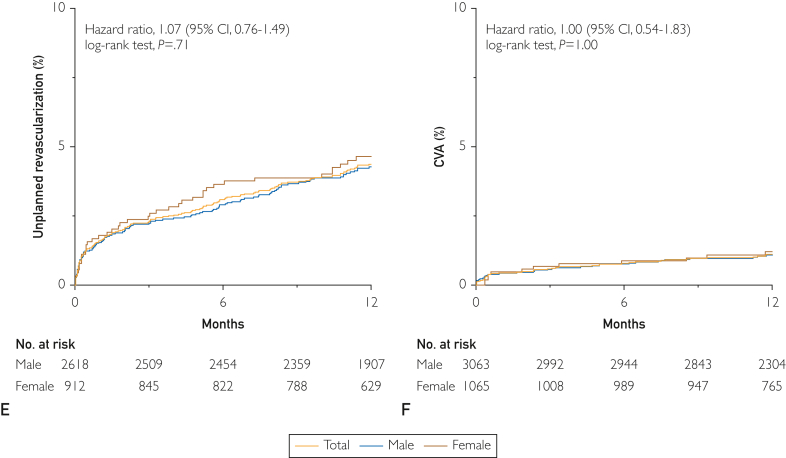
Sex-based cardiovascular outcomes postrevascularization. A, Cumulative incidence of major adverse cardiovascular events (myocardial infarction [MI], unplanned revascularization, death, cerebrovascular accident [CVA]). Subsequent panels demonstrating cumulative incidence of individual components including (B) MI and unplanned revascularization, (C) mortality, (D) MI, (E) unplanned revascularization, and (F) CVA in the year following revascularization. Total cohort (**blue**), male patients (**red**), female patients (**green**) with hazard ratios (HRs) and 95% confidence intervals (CIs) presented for outcomes of women compared with men.

The median follow-up period for the full cohort was 13.3 months (IQR: 11.9-16.5 months). During this study period, MACE occurred in 11.0%, death in 5.6%, MI in 1.7%, unplanned revascularization in 4.2%, and cerebrovascular accidents in 1.4%; MACE occurred in 3.9% of patients at 30 days. (Figure 3). Female patients demonstrated higher rates of MACE than male patients, driven primarily by greater rates of death (HR, 1.90; 95% CI, 1.46 to 2.47; *P*<0.0001). No differences in MACE were observed between patients undergoing PCI or CABG (11.3% vs 9.8%; HR, 1.20 [0.92 to 1.58]; *P*=0.18). Subgroup analysis of patients who presented as ACS vs stable CAD demonstrated a higher proportion of MACE in the ACS cohort (12.5% vs 7.5%; HR, 1.73 [1.34-2.24]; *P*<.0001) (Supplemental Figure 1, available online at http://mcpiqojournal.org). Patients with 3-vessel disease accrued higher MACE rates in follow-up, an effect that remained consistent in both men and women, although with a trend toward women experiencing greater rates of MACE in the setting of 3-vessel disease (Supplemental Figure 2, available online at http://mcpiqojournal.org). Similarly, worsening left-ventricular function also portended greater rates of MACE in follow-up (Supplemental Figure 3, available online at http://mcpiqojournal.org). Unadjusted and adjusted analysis did not suggest that female sex was not associated with MACE (HR, 1.21 [0.97-1.52] or death (HR, 1.30 [0.96-1.75]) in the year following revascularization (Supplemental Table 1, available online at http://mayoclinicproceedings.org).

### Risk-Factor Management

**Figure 4:**
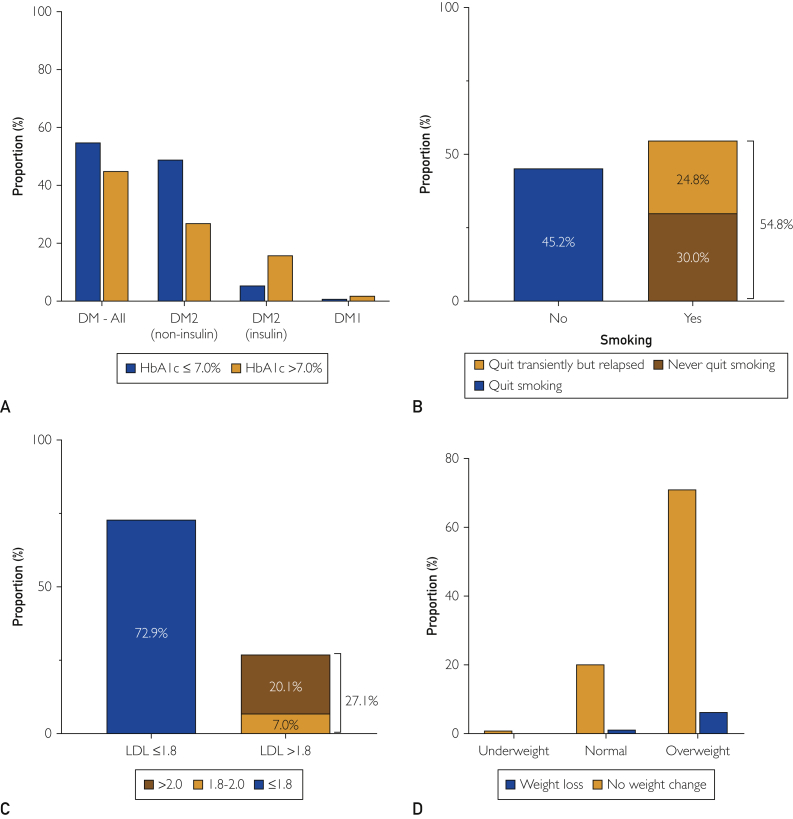
Risk factor management at 1 year. A, Glycemic control defined as HbA1c ≤ 7.0% was achieved in 55.0% of patients. Of the 335 patients with diabetes who failed to achieve target HbA1c at 1 year (45.0%), 14 (1.9%) had type 1 diabetes, 202 (27.1%) type 2 diabetes (non–insulin-dependent), and 119 (16%) had type 2 diabetes (insulin-dependent). B, Baseline active smokers were assessed at the time of follow-up for smoking cessation; 45.2% had quit smoking in follow-up, whereas 54.8% continued to smoke (24.8% having quit but relapsed, 30.0% having never quit). C, Lipid control was defined as LDL ≤ 1.8 mmol/L; 137 patients (7.0%) had low-density lipoprotein (LDL) levels between 1.8 and 2.0 mmol/L, and 393 patients (20.1%) had LDL > 2.0 mmol/L. D, Weight loss >10% was achieved at follow-up in 175 patients (6.3%) who were overweight or obese and in 31 patients (1.1%) with normal weight (D).

Hemoglobin (Hb) A1c was available in 745 (68.3%) patients with DM at 1-year follow-up with 410 patients (55.0%) achieving adequate glycemic control (HbA1c ≤ 7.0%). Of the 335 patients with DM who failed to achieve target HbA1c at 1 year (45.0%), 14 (1.9%) had type 1 diabetes, 202 (27.1%) type 2 diabetes (non–insulin-dependent), and 119 (16%) had type 2 diabetes (insulin-dependent) (Figure 4A). Smoking status was assessed and documented in 4004 patients, 3574 patients of whom (89.3%) were not smoking at the time of clinical follow-up. Among the nonsmokers, 1889 patients (47.2%) were lifelong nonsmokers, and 1286 patients (32.1%) quit before the index procedure. Among active smokers at the time of angiography, 45.2% had quit smoking at follow-up, whereas 54.8% continued to smoke (24.8% having quit but relapsed, 30.0% having never quit) (Figure 4B). Lipid levels were available in 1955 patients (47.1%). A total of 1425 patients (72.9%) achieved an LDL ≤1.8 mmol/L, 137 patients (7.0%) had LDLs level between 1.8 and 2.0 mmol/L, and 393 patients (20.1%) had LDL >2.0 mmol/L (Figure 4C). At follow-up, 84% of all patients were on statins, with female patients less likely than male patients to be on statins (81% vs 85%, *P*=0.002) (Table). In those failing to achieve LDL targets, 5% were not on statins. Body mass index was recorded in 3484 patients at the time of the index procedure and at 1 year in 2762 patients; 2145 patients (77.7%) were overweight or obese, 594 patients (21.5%) were normal weight, and 23 patients (0.8%) were underweight. Weight loss >10% was achieved at follow-up in 175 patients (6.3%) who were overweight or obese and in 31 patients (1.1%) with normal weight (Figure 4D). Women were less likely to achieve target LDL and smoking cessation in follow-up compared with men (Supplemental Figure 4, available online at http://mcpiqojournal.org).

### Modifiable Risk-Factor Burden

**Figure 5:**
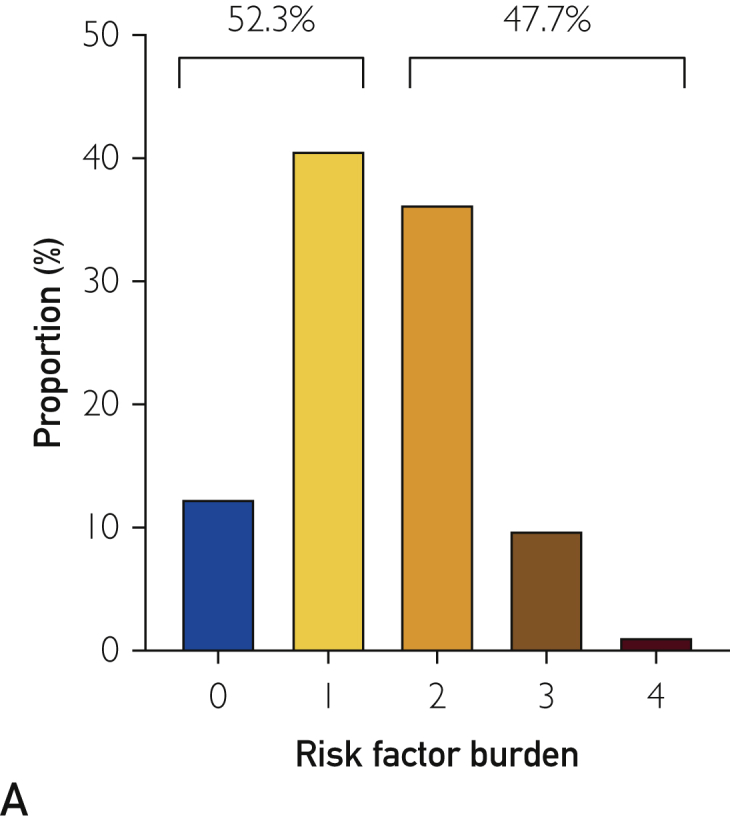

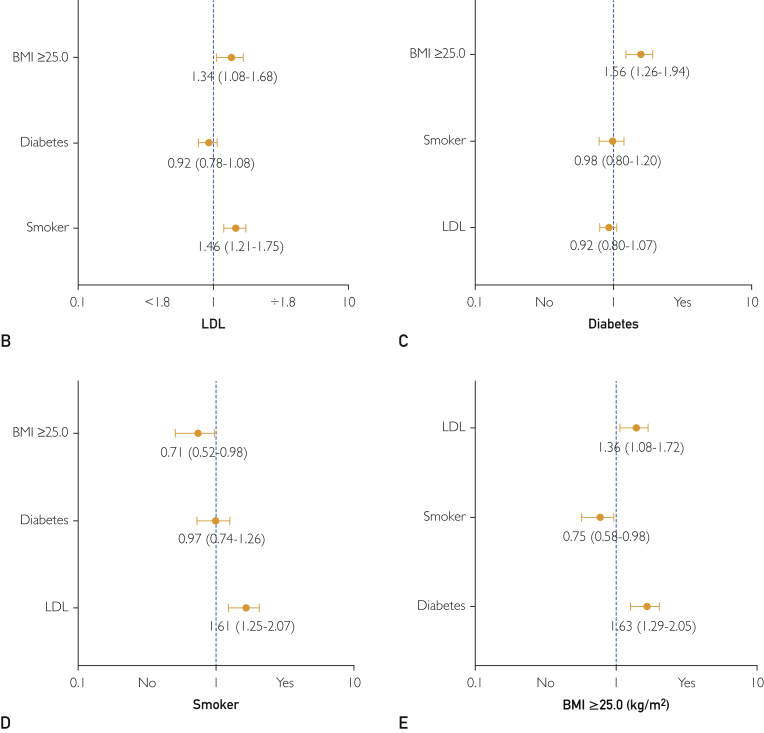
Modifiable risk factor burden. A, Burden of modifiable risk factors displayed on a patient-level and dichotomized to those patients with ≥ 2 (47.7%) or <2 (52.3%) risk factors. B-E, Interactions among risk factors were noted, with overweight patients being more likely to have diabetes (odds ratio [OR], 1.56; 95% confidence interval [CI], 1.26 to 1.94), whereas both overweight patients (OR, 1.34; 95% CI, 1.08 to 1.68) and smokers (OR, 1.46; 95% CI, 1.21 to 1.75) were more likely to have LDL >1.8 mmol/L.

At 1-year follow-up, 47.7% of patients had ≥2 modifiable risk factors identified (Figure 5A). Associations between risk factors were noted with overweight patients more likely to have DM (OR, 1.56; 95% CI, 1.26 to 1.94) and to have LDL ≥1.8 mmol/L (OR, 1.34; 95% CI, 1.08 to 1.68), whereas active smokers were more likely to have LDL ≥1.8 mmol/L (OR, 1.46; 95% CI, 1.21 to 1.75) at 1 year (Figure 5B-E). Subgroup analysis of individual risk factors demonstrated that patients with DM had markedly elevated rates of MI and repeat revascularization at 1 year (HR, 1.84; 95% CI, 1.40 to 2.42; *P*<.0001) (Supplemental Figure 5, available online at http://mcpiqojournal.org).

## Discussion

**Figure 6:**
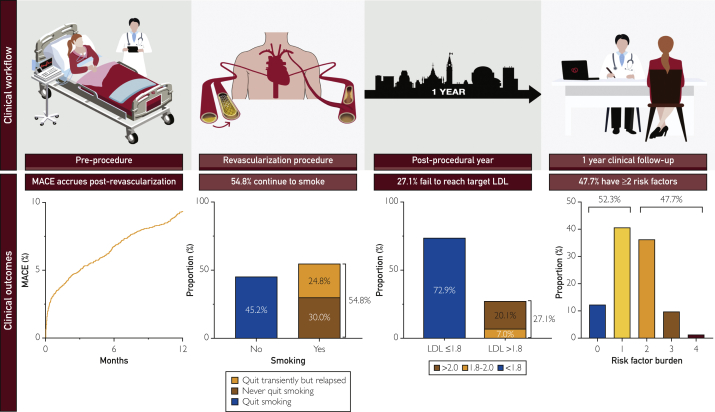
Clinical workflow of postrevascularization clinic including preprocedure, revascularization procedures, postprocedural year ,and 1-year clinical follow-up. Summarized clinical outcomes over that time include continual accruing major adverse cardiovascular events, one-half of smokers failing to quit, and one-quarter failing to reach target lipid levels, whereas one-half of patients have ≥2 cardiovascular risk factors.

We established a postrevascularization program to standardize clinical follow-up of patients in the year following revascularization, with the goal of optimizing risk-factor management and implementing secondary prevention strategies. Our real-world experience demonstrates several important points. First, significant residual risk exists in this cohort with nearly 1 in 10 patients experiencing MACE in the first 12 months and female patients being disproportionately at risk. Second, there is a high prevalence of uncontrolled risk factors, with one-quarter not achieving target LDL, one-half of smokers continuing to smoke, and one-half of patients with DM not achieving target HbA1c. Finally, risk-factor clustering is common, with one-half of patients having ≥2 factors (Figure 6). Overall, these data highlight that the postrevascularization patient is at high risk for adverse events, with a significant number of patients failing to achieve optimal risk-factor management during their highest-risk period.

Established cardiovascular risk factors are known to increase the risk of adverse events, thus motivating targeted interventions.^1^ Unfortunately, risk-factor burden often does not translate into behavioral changes, including among patients at highest cardiac risk.^39^ Cardiac rehabilitation with focused risk-factor management programs have made considerable strides in this regard.^40^ Similarly, dedicated diabetes-management programs can enhance glycemic control, translating to improved cardiovascular outcomes.^41^^,^^42^ Smoking-cessation programs can be similarly effective.^43^ Our center previously developed the Ottawa Model for Smoking Cessation, which has yielded cessation rates of up to 44% to 61% at 6 months and improved outcomes.^23^^,^^24^^,^^44^ Yet, despite these and other programs being in place at our center, in our study, many patients failed to modify their cardiovascular risk factors adequately. This includes the inability to achieve target HbA1c, weight loss, or cessation of smoking in follow-up, in part highlighting the difficult nature of modifying patient behavior.^39^ Health care providers may also be contributing to suboptimal risk- factor control by undertreating certain patients. For instance, lipid control has been well established as a strategy to reduce cardiovascular risk,^45^^,^^46^ with current guidelines suggesting maximal-dose statin therapy and treating to an LDL target of 1.8 to 2.0 mmol/L, depending, in part, on the clinical presentation.^34^, ^35^, ^36^ In our cohort, one-quarter of patients failed to achieve guideline-directed targets (7% with LDL 1.8 to 2.0 and 20.1% with LDL > 2.0), including 5% who were not on any statin therapy at follow-up. Female patients were disproportionately affected, being less likely to achieve target LDL and be on statin therapy, in keeping with previous reports.^47^ Taken together, these findings highlight potential gaps in reduction of cardiovascular risk in routine postrevascularization care.

Accurately predicting the risk of adverse events postrevascularization is important for guiding targeted follow-up and therapies. Patients remain at high risk of adverse events postrevascularization, with women disproportionately afflicted by elevated risk of death and MACE.^48^ The precise etiology for these disparities remains unclear, with some postulating that despite presenting with less extensive disease (ie, non–3-vessel disease), women may carry a more aggressive CAD phenotype.^48^ Without a doubt, the importance of monitoring for sex-specific differences remains and cannot be understated, similar to other cardiovascular interventions.^49^ Despite this, cardiovascular trials continue to report declining rates of female enrollment,^50^^,^^51^ prompting calls for improved methodological rigor,^52^ with strategies including standardized checklists^53^ to improve sex-specific outcome reporting.

Indeed, the additive impact of cumulative risk factors has been previously discussed.^54^ In our study, risk-factor clustering was observed. The significance of this phenomenon is clear when one considers that patients with DM are known to have elevated cardiovascular risk,^55^ but, when combined with additional risk factors, their risk of mortality doubles.^56^ In our study, we demonstrate that one-half of patients have 2 or more risk factors and may influence each other. For instance, overweight individuals were more likely to have DM and were less likely to achieve target LDL. Smokers were similarly less likely to achieve target LDL than nonsmokers. Some of these associations may be physiological in nature, whereas others may reflect underlying behavioral tendencies. Regardless, identifying high-risk patients, particularly those for whom intervention may be beneficial, could improve postrevascularization care and focus efforts and resources on efforts with greatest likelihood of impact.

### Limitations

Our data are subject to selection bias, including survival bias, in that the risk factors of patients who were lost to follow-up or who died before their planned follow-up are unknown. However, patients who return for follow-up are likely to be more adherent with medical therapy and have improved risk-factor management. Owing to selection and survival bias, our study potentially overestimates the effectiveness of current postrevascularization-care strategies of secondary prevention. Our report is limited to the first year postrevascularization, while known to be the highest risk period in this patient population, long-term insights are limited. Although our established cardiac-rehabilitation program is offered to all revascularization patients, a detailed assessment of this was beyond the scope of this study. Therefore, our findings highlight important areas for potential improvement in patient care postrevascularization. Heightened and focused efforts on early and sustained cardiovascular risk reduction in this patient population are warranted.

## Conclusion

Patients who have undergone coronary revascularization are at high risk of MACE and often have suboptimally managed modifiable risk factors at 1 year post-procedure. Targeted efforts to identify this subset of patients and to reduce their risk of future cardiovascular events effectively should be prioritized. Optimal follow-up pathways must be established to maximize the clinical benefits of revascularization.
