# Supplementary material for: Modifiable Risk Factors and Residual Risk Following Coronary Revascularization: Insights From a Regionalized Dedicated Follow-Up Clinic
Source: Mayo Clin Proc Innov Qual Outcomes. 2021 Dec 4;5(6):1138–52. doi: 10.1016/j.mayocpiqo.2021.09.001 (PMC8654638; doi:10.1016/j.mayocpiqo.2021.09.001)
Supplement: Supplemental Data [file mmc1.pdf]

Supplemental Files

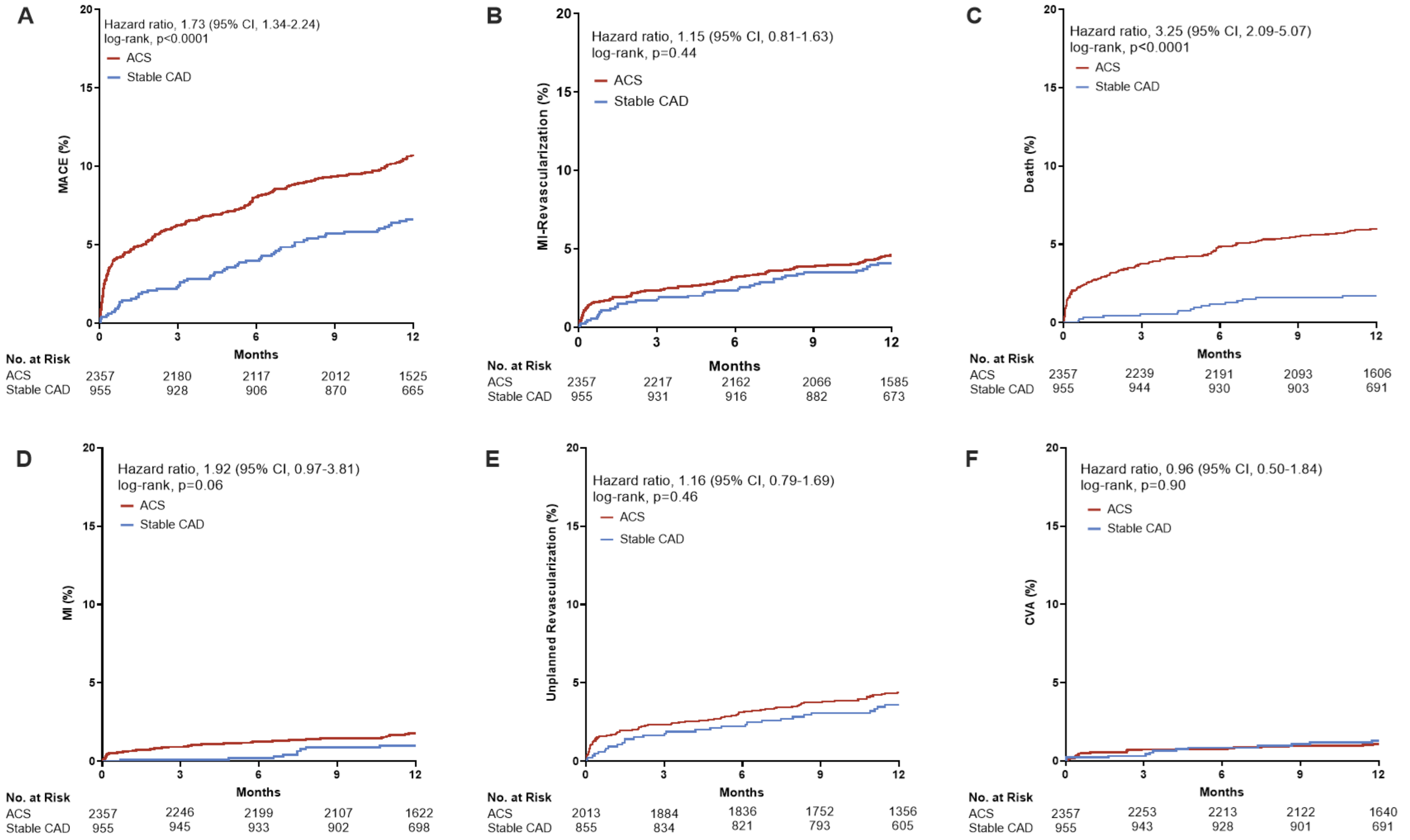

Supplemental Figure 1. Impact of acute coronary syndrome (ACS) on clinical outcomes. ACS patients noted to have elevated rates of MACE (A) and death (C) when compared to stable CAD controls, no differences in MI-revascularization (B), MI (D), revascularization (E), or CVA (F).

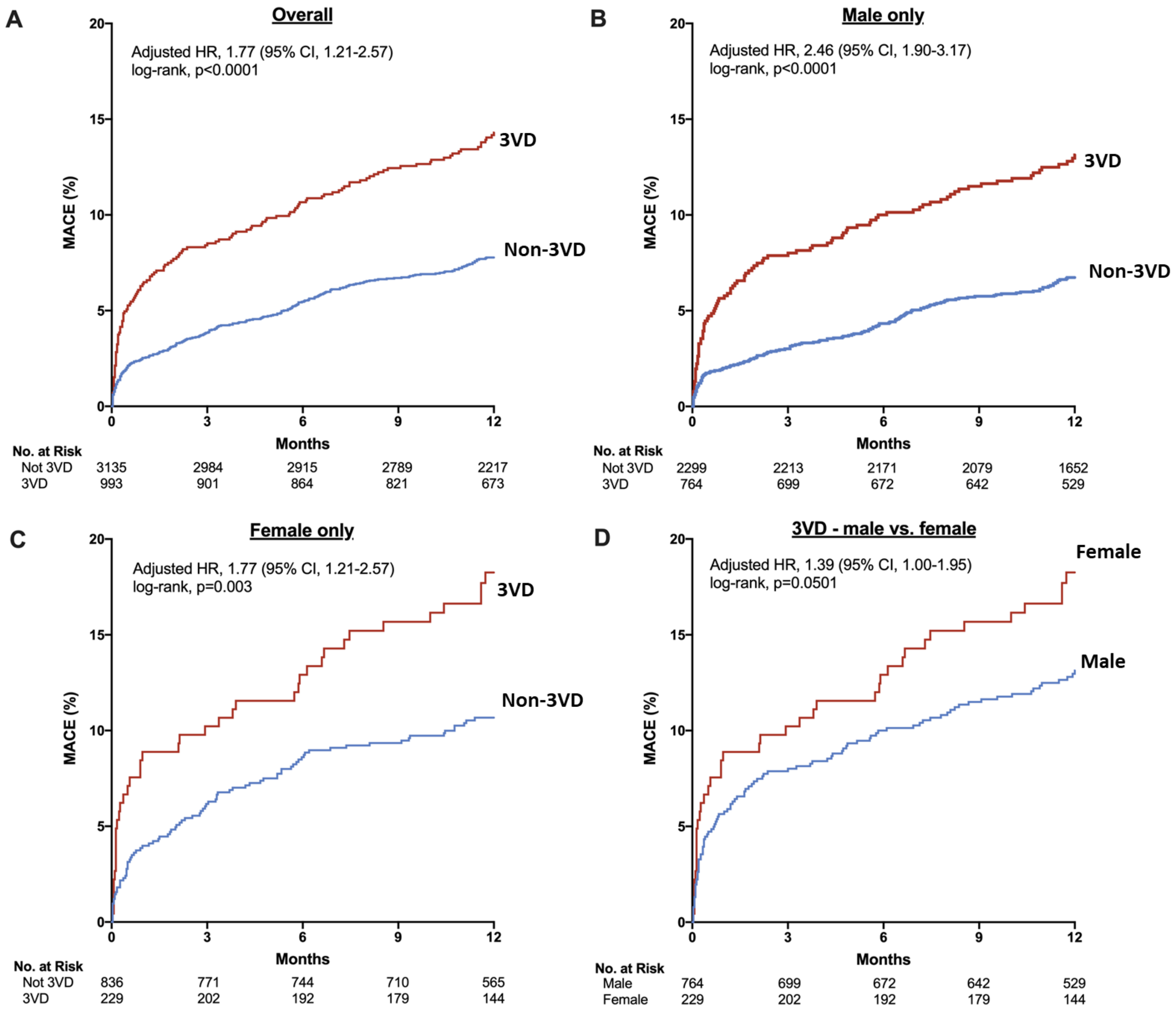

Supplemental Figure 2. Sex-based impact of coronary artery disease (CAD) burden on clinical outcomes. **(A)** Total cohort dichotomized into those with (3VD) or without three-vessel CAD (non-3VD). Sex-based differences of the impact of 3VD vs non-3VD assessed in males **(B)** and females **(C). (D)** When assessing strictly those with 3VD females demonstrate more adverse events than males. MACE – major adverse cardiac events - death, myocardial infarction, cerebrovascular accident, unplanned revascularization. Hazard ratios (HR) and 95% confidence intervals (CI) presented for outcomes of 3VD compared to non-3VD and females compared to males.

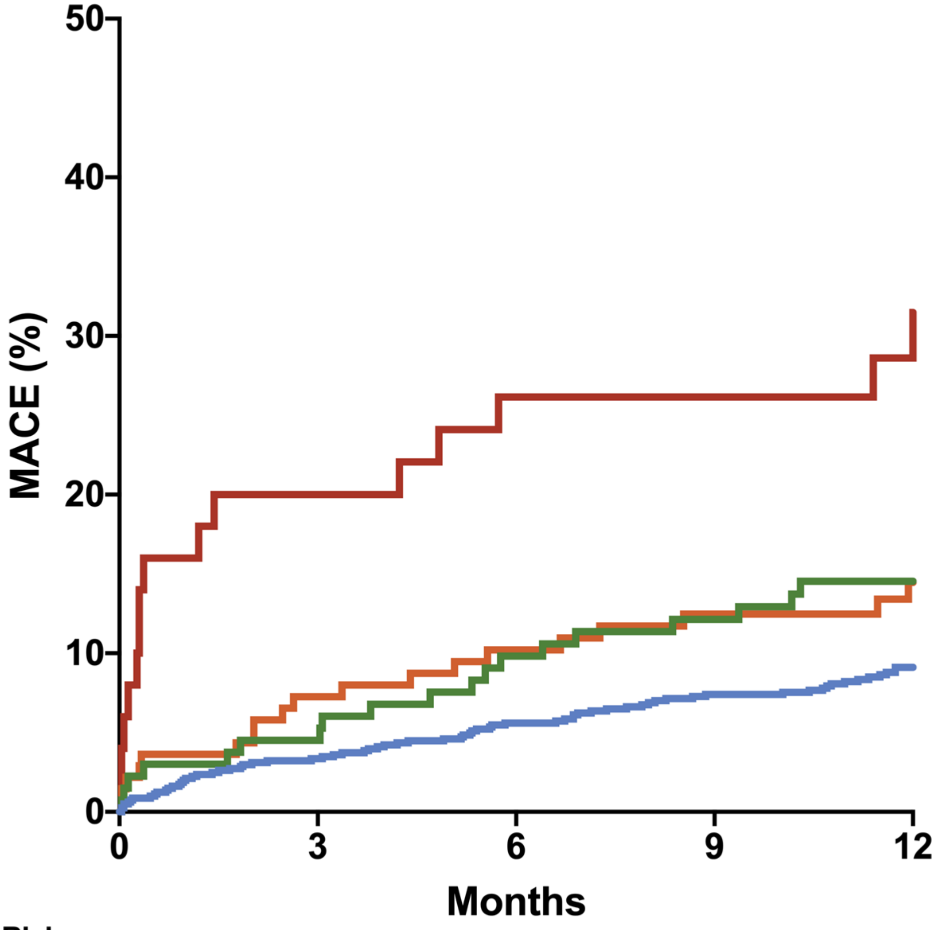

No. at Risk

Normal	810	776	749	709	551
≥ 45%	133	126	118	113	90
30-45%	138	127	121	112	81
< 30%	51	39	36	34	24

Supplemental Figure 3. Impact of left ventricular function on clinical outcomes. Clinical outcomes stratified by left ventricular function on presentation (<30% -red, 30-45%-orange, ≥45%-orange**,** ≥55%-blue) demonstrating deteriorating clinical outcomes with worsening LV function. MACE – major adverse cardiac events - death, myocardial infarction, cerebrovascular accident, unplanned revascularization.

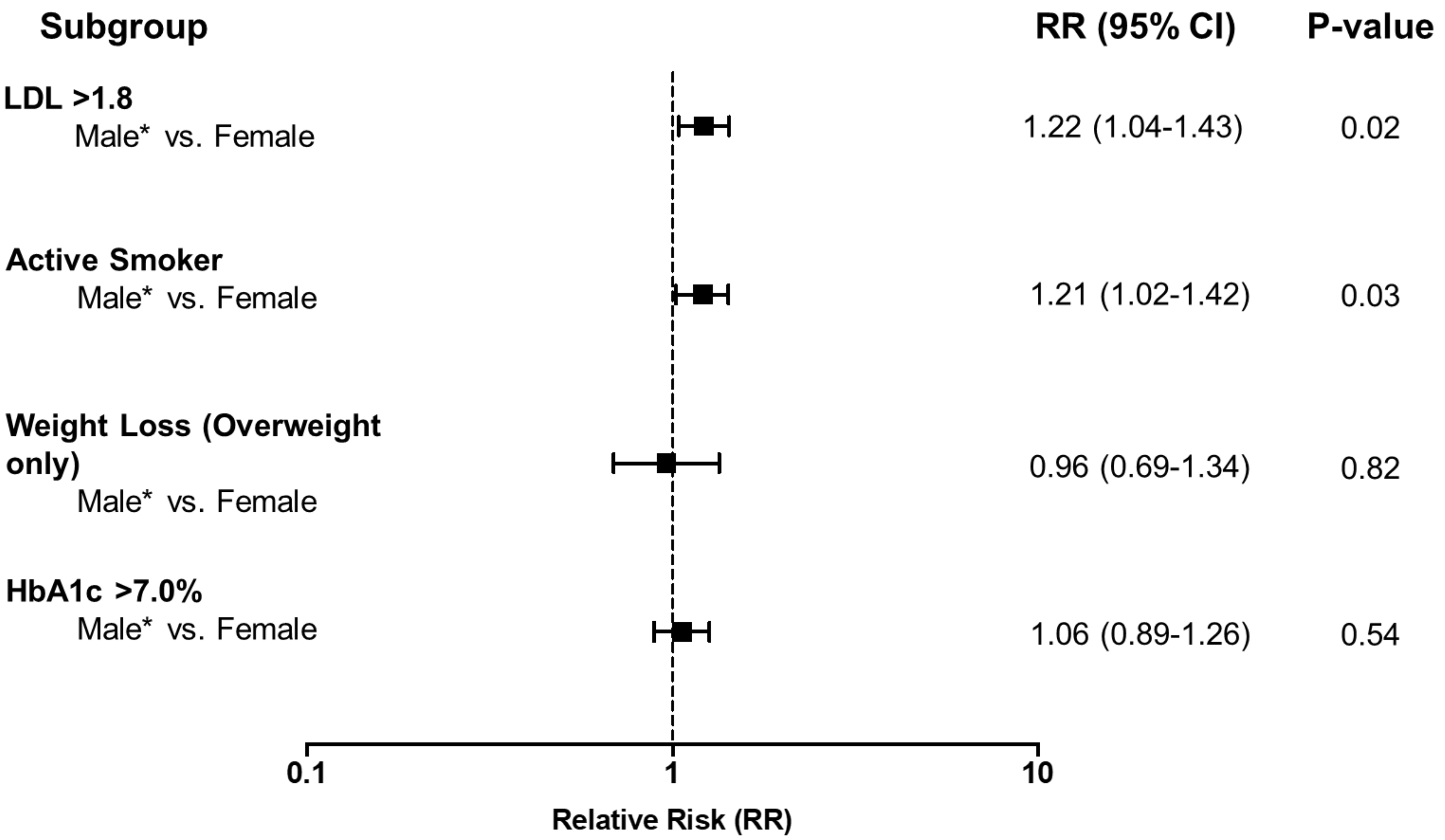

Supplemental Figure 4. Sex-based differences in optimal risk factor management. Subgroup analysis of risk factor control stratified by sex. Unadjusted relative risk of respective risk factor management with 95% confidence interval are shown above. Each subgroup was dichotomized and the reference group is denoted with an asterisk. P-value less than 0.05 was considered statistically significant. LDL – low density lipoprotein.

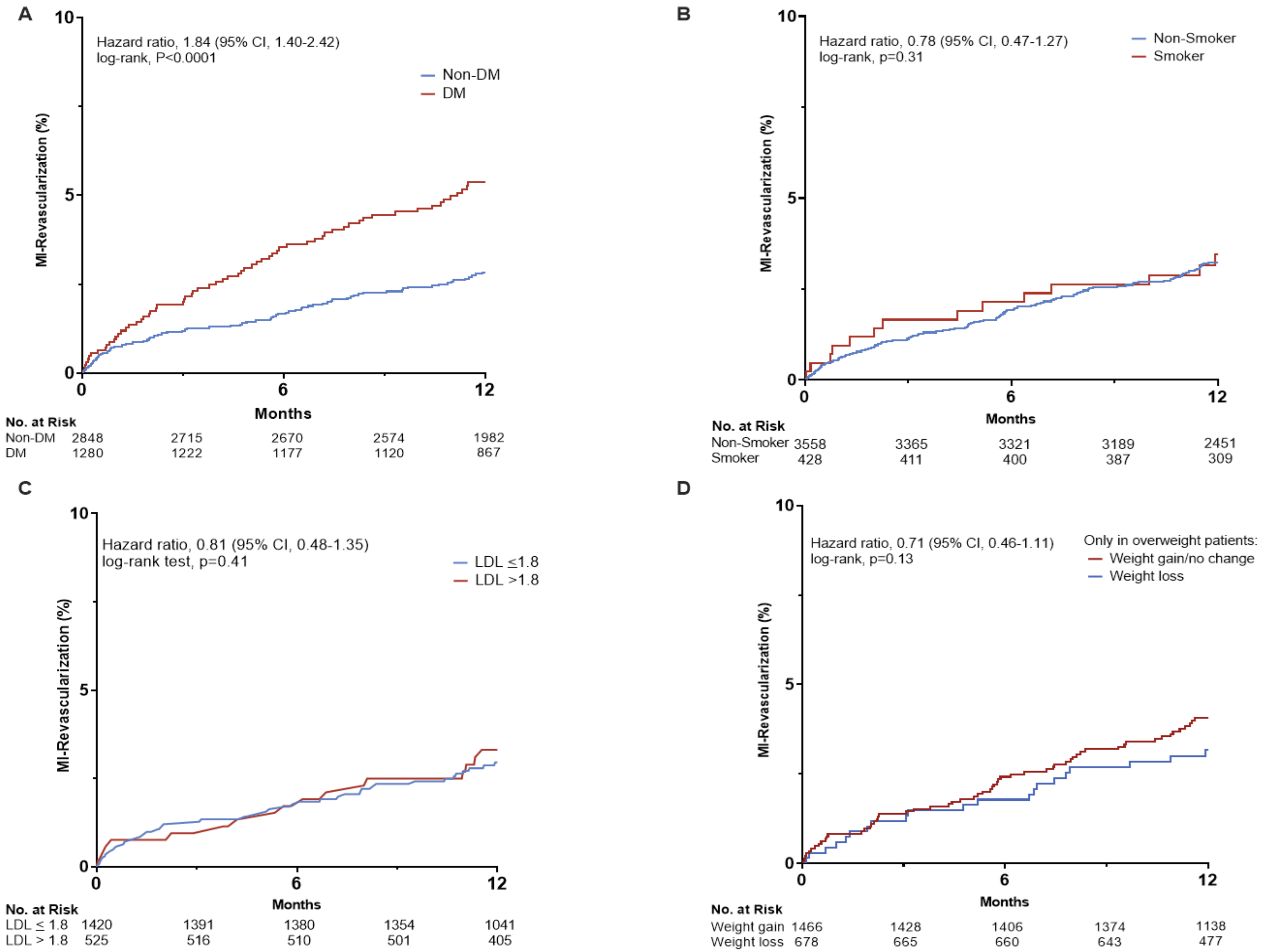

Supplemental Figure 5. Association of modifiable risk factors with clinical outcomes

(A) Patients with diabetes had elevated rates of myocardial infarction and unplanned revascularization (MI-revascularization) (hazard ratio, 1.84; 95% CI, 1.40 to 2.42; p<0.0001). **(B)** Active smoking was not associated with increased rates of MI-revascularization (hazard ratio, 0.78; 95% CI, 0.47 to 1.27; p=0.31). **(C)** LDL control ≤1.8 mmol/L was not associated with MI-revascularization (hazard ratio, 0.81; 95% CI, 0.48 to 1.35; p=0.41). **(D)** Weight loss in overweight patients was not associated with MI-revascularization (hazard ratio, 0.71; 95% CI, 0.46 to 1.11; p=0.13). Kaplan-Meier curves were generated and compared by log-rank test and hazard ratios were evaluated using the Cox proportional hazards model. P<0.05 is considered statistically significant.

Supplemental Table 1. Clinical outcomes associated with gender

	Unadjusted HR (95% CI)	Adjusted HR (95% CI)*
**MACE**	1.52 (1.25-1.85)	1.21 (0.97-1.52)
**Death**	1.90 (1.46-2.47)	1.30 (0.96-1.75)

Reference for gender is male

*Adjusted for age, acute coronary syndrome, type 2 diabetes, active smoking, dyslipidemia, hypertension, congestive heart failure, and obesity
